# The effects of treatment with atorvastatin versus rosuvastatin on endothelial dysfunction in patients with hyperlipidaemia

**DOI:** 10.5830/CVJA-2018-008

**Published:** 2018

**Authors:** Demir Vahit, Ede Hüseyin, Tolga Doğru Mehmet, Alp Cağlar, Celik Yunus, Yıldırım Nesligül, Yılmaz Samet

**Affiliations:** Cardiology Department, Faculty of Medicine, Bozok University, Yozgat, Turkey; Cardiology Department, Faculty of Medicine, Bozok University, Yozgat, Turkey; Cardiology Department, Faculty of Medicine, Kırıkkale University, Kırıkkale, Turkey; Cardiology Department, Faculty of Medicine, Kırıkkale University, Kırıkkale, Turkey; Cardiology Department, Faculty of Medicine, Kırıkkale University, Kırıkkale, Turkey; Cardiology Department, Faculty of Medicine, Kırıkkale University, Kırıkkale, Turkey; Cardiology Department, Yozgat State Hospital, Yozgat, Turkey

**Keywords:** atorvastatin, endothelial function, flow-mediated vasodilatation, rosuvastatin

## Abstract

**Introduction:**

Statins can reduce cardiovascular events and improve endothelial function. However, differences in the effect of statins on endothelial dysfunction have not been researched sufficiently. Here, we aimed to compare the effects of atorvastatin versus rosuvastatin on endothelial function via flow–mediated and endothelial–independent dilation.

**Methods:**

Hyperlipidaemic subjects on treatment with statins for one year (either 20 mg/day atorvastatin or 10 mg/day rosuvastatin) were enrolled in the study. In accordance with the literature, flow–mediated dilation (FMD) and nitrate–mediated endothelium–independent dilation (EID) were measured by ultrasonography on the right brachial artery of each subject. Baseline and final measurements were compared in each group and between the groups.

**Results:**

One hundred and four subjects (50 atorvastatin and 54 rosuvastatin users) were enrolled in the study. Fifty–eight subjects were female. The groups were statistically similar in terms of age and body mass index, and haemoglobin, creatinine, total cholesterol, triglyceride, high–density lipoprotein and low–density lipoprotein cholesterol levels. In each group, the mean final FMD and EID values were higher compared to their respective baseline values, but the mean changes in FMD and EID were statistically similar in both groups (p = 0.958 for FMD and 0.827 for EID). There was no statistically significant difference between the atorvastatin and rosuvastatin groups in terms of final FMD and EID values (p = 0.122 and 0.115, respectively).

**Conclusion:**

This study demonstrated that both one–year atorvastatin and rosuvastatin treatments significantly improved endothelial function, when assessed with FMD and EID and measured by ultrasonography. However, the amount of improvement in endothelial dysfunction was similar in the two treatments.

Hyperlipidaemia is an important risk factor for the development of atherosclerosis. Statins may reduce the risk of cardiovascular events and improve endothelial function.^1^,^2^ The positive effect of statins on endothelial dysfunction is carried out via endothelial nitric oxide enzyme activation.^3^ Pleiotropic effects of statins include improvement in endothelial function, anti–thrombosis, plaque stabilisation and anti–oxidative effects, and decreasing the duration of vascular inflammation.^4^ However, differences in the effect of statins on endothelial dysfunction has not been researched sufficiently. Earlier studies demonstrated that improved endothelial dysfunction in different vascular beds started after a few days of treatment with statins.^5^–^8^

Endothelial dysfunction is the early sign of atherosclerosis and enhances the risk of cardiovascular events.^9^ Flow–mediated dilation (FMD) is a well–known method used for predicting the extent of atherosclerosis. FMD is measured on the brachial arteries and reflects the ability of an artery to enlarge after being compressed for a certain time. Nitric oxide (NO) is the most important vascular vasodilator and is produced by the endothelium in response to certain factors such as shear stress. Its production is impaired in the case of endothelial dysfunction. Increased production of NO after increased vascular blood flow is the underlying mechanism of FMD.^10^

Studies on primary and secondary prevention of cardiovascular disease and its complications by statins revealed that their effect occurs not only due to their lipid–lowering effect but also due to pleiotropic effects, the mechanism of which remains unclear. In this study we aimed to compare the effect of one–year rosuvastatin versus atorvastatin therapy on endothelial function in hyperlipidaemic patients, using FMD and endotheliumindependent dilation (EID), measured ultrasonographically on the brachial artery.

## Methods

A total of 112 patients diagnosed with hyperlipidaemia and without a history of previous lipid–lowering medication for at least the previous two months, and with an indication for medical treatment despite a first–line, four–week, lipid–lowering diet, applied to the cardiology out–patient unit and were enrolled in the study between May 2010 and August 2011. Approval of the local ethics committee and informed consents of the participants were obtained accordingly.

A subject was considered treatment adherent when he/she took her/his prescribed statin regularly on a daily basis. A subject was considered complient if his/her baseline and post–treatment measurements were obtained as per the study protocol. Eight cases (four with compliance problems with follow up, one with lung cancer and three with non–adherence to medication) were excluded from the study.

The study was completed with 104 hyperlipidaemic patients, of whom 50 were assigned to atorvastatin 20 mg per day and 54 to rosuvastatin 10 mg per day. Patients under the age of 18 and over the age of 80 years, those with heart failure, uncontrolled hypertension, endocrine diseases, previous coronary artery disease, frequent and permanent cardiac dysrhythmia, malignancy, chronic obstructive pulmonary disease, and chronic liver, kidney, neurological or psychiatric diseases, which were likely to produce a compliance problem, were not included in the study.

Baseline demographic characteristics of the patients were recorded. Body mass index (BMI) was calculated as body weight (kg)/height^2^ (m). Levels of fasting blood glucose, serum total cholesterol, high–density lipoprotein (HDL) cholesterol, low–density lipoprotein (LDL) cholesterol, triglycerides (TG), urea, creatinine, aspartate transaminase (AST), alanine transaminase (ALT), creatinine phosphokinase (CPK) and complete blood counts were measured in all patients after a 12–hour fasting period. In addition, the patients underwent transthoracic echocardiography.

Lipid levels indicated eligible patients, who were randomly assigned to receive either rosuvastatin 10 mg/day or atorvastatin 20 mg/day. The patients were followed for one year. Baseline measurements were repeated at the end of the 12–month treatment period. Change in LDL level (ΔLDL) was defined as the difference between baseline and post–treatment LDL values.

Endothelial function was measured ultrasonographically over the brachial artery using echocardiography (Ge–Vivid 7 Pro, General Electric, Florida, USA) with a 12–L probe. All measurements were performed according to the method described elsewhere in the literature.^11^ Brachial artery basal Doppler velocity (DV), basal diameter (BD), brachial artery hyperaemia velocity (HV), and post–flow brachial artery lumen diameter (hyperaemia diameter = HD flow–mediated dilation response = FMDR) were recorded. FMD was calculated from the following equation:

% FMD = FMDR – BD/BD * 100

Baseline endothelium–independent dilation (EID) was measured 10 minutes after deflation of the cuff to obtain baseline conditions and was labelled as pre–nitrate BD. Thereafter, the patients received 400 μg of nitroglycerin sublingually; three to five minutes later, post–nitrate Doppler, post–nitrate velocity (NTGV) and post–nitrate arterial diameter (NTGD) were measured. Lumen diameter was measured three times and the arithmetic mean was calculated. Post–nitrate arterial diameter was named nitrate–mediated dilation response (NMDR). EID was calculated using the following equation:

% EBG = NMDR – pre-nitrate BD/Pre-nitrate BD * 100

ΔFMD and ΔEID were defined as the difference between baseline and post–treatment FMD and EID values, respectively.

## Statistical analyses

The SPSS (SPSS, Inc, Chicago, IL, USA) program was used to analyse the data. Mean and standard deviations (SD) were used for descriptive data. Student’s t–test was used to compare normally distributed quantitative variables, whereas the Mann– Whitney U–test was used to compare independent non–normally distributed quantitative variables. Moreover, statistical comparison between continuous dependent variables was done by paired–samples t–test for normally distributed variables, whereas the Wilcoxon test was used for non–normally distributed variables. Relationships between the parameters were assessed with Pearson’s correlation analysis for parametric variables and by Spearman’s correlation analysis for non–parametric variables. The results were evaluated with a 95% confidence interval and at the significance level of p < 0.05.

## Results

A total of 104 hyperlipidaemic cases were included in the study. The patients were randomly assigned to either atorvastatin (group 1, n = 50, 48.1%) or rosuvastatin (group 2, n = 54, 51.9%) therapy. Of the overall patients, 46 were male (53.7 ± 9.7 years) and 58 were female (54.3 ± 10.1 years). There was no statistically significant difference between the two groups in terms of baseline anthropometric characteristics of the subjects, haemoglobin, haematocrit, white blood cell count, thrombocyte count, and urea, creatinine, AST, ALT, CPK, total cholesterol, TG, HDL and LDL levels.

Mean ΔLDL at the end of 12 months was 71.0 ± 29.7 mg/ dl (1.84 ± 0.77 mmol/l) and percentage ΔLDL was 42.2 ± 17.6% (n = 104) in the study population. ΔLDL was significantly correlated with ΔFMD (r = 0.367, p < 0.005) and ΔEID (r = 0.523, p < 0.001). Percentage ΔLDL was statistically correlated with ΔFMD (r = 0.412, p < 0.005) and ΔEID (r = 523, p < 0.001). In the atorvastatin group, a statistically significant reduction was shown in total cholesterol, LDL and TG levels compared to baseline values. LDL level showed a 52.5% decrease after 12 months compared to baseline value, whereas no decrease was observed in HDL level. FMD showed a statistically significant increase (Table 1).

**Table 1:** Post-treatment versus baseline values in the atorvastatin group

*Atorvastatin*	*Baseline mean ± (SD)*	*12-month mean ± (SD)*	p-value*
Basal diameter (mm)	4.0 ± 0.6	4.1 ± 0.6	0.045
Hyperaemia diameter (mm)	4.2 ± 0.6	4.3 ± 0.6	0.436
NTG diameter (mm)	4.5 ± 0.6	4.7 ± 0.6	0.002
FMD (%)	8.5 ± 3.3	10.4 ± 4.1	< 0.001
EID (%)	15.5 ± 5.1	16.3 ± 4.8	0.143
TC (mg/dl)	261.3 ± 28.3	174.3 ± 38.9	< 0.001
(mmol/l)	(6.77 ± 0.73)	(4.51 ± 1.01)	
TG (mg/dl)	161.8 ± 66	131.9 ± 50	< 0.001
(mmol/l)	(1.83 ± 0.75)	(1.49 ± 0.57)	
LDL-C (mg/dl)	176.8 ± 23.5	92.9 ± 28.1	< 0.001
(mmol/l)	(4.58 ± 0.61)	(2.41 ± 0.73)
HDL-C (mg/dl)	54.7 ± 12.1	54.4 ± 12.4	0.145
(mmol/l)	(1.42 ± 0.31)	(1.41 ± 0.32)	
AST (U/l)	23.8 ± 9.1	21.9 ± 5.6	0.068
ALT (U/l)	23.6 ± 12.8	24.1 ± 13.1	0.746
CPK (U/l)	136.8 ± 74	113.4 ± 67	0.427

*Student’s t-test, p < 0.05.

SD: standard deviation; NTG: post-nitrate; FMD: flow-mediated dilation; EID: endothelium-independent dilation; AST: aspartate transaminase; ALT: alanine transaminase; CPK: creatinine phosphokinase; TG: triglycerides; TC: total cholesterol; LDL-C: low-density lipoprotein cholesterol; HDL-C: high-density lipoprotein cholesterol.

In the rosuvastatin group, a statistically significant decrease was found in total cholesterol, LDL and TG levels compared to baseline values. LDL level showed a 58.5% decrease at the end of the 12 months compared to baseline value, whereas no change was observed in HDL levels. While a statistically significant increase was observed in the brachial artery basal diameter and hyperaemia diameter compared to baseline values, no change was observed in the post–nitrate diameter and EID values. FMD showed a statistically significant increase compared to baseline (Table 2).

**Table 2:** Post-treatment versus baseline values in the rosuvastatin group

*Rosuvastatin*	*Baseline*	*12 months*	p-value*
Basal diameter (mm)	4.0 ± 0.5	4.2 ± 0.5	0.003
Hyperaemia diameter (mm)	4.4 ± 0.5	4.6 ± 0.5	< 0.001
NTG diameter (mm)	4.6 ± 0.5	4.7 ± 0.5	0.687
FMD (%)	9.7 ± 3.4	12.7 ± 3.7	< 0.001
EID (%)	16.8 ± 5.8	18.2 ± 5.8	0.105
TC (mg/dl)	271.2 ± 35.7	188.4 ± 44.8	< 0.001
(mmol/l)	(7.02 ± 0.92)	(4.88 ± 1.16)	
TG (mg/dl)	173.5 ± 55.2	143 ± 54.1	< 0.001
(mmol/l)	(1.96 ± 0.62)	(1.62 ± 0.61)	
LDL-C (mg/dl)	180.5 ± 26.1	105 ± 39.2	< 0.001
(mmol/l)	(4.67 ± 0.68)	(2.72 ± 1.02)	
HDL-C (mg/dl)	56.8 ± 13.6	54 ± 11.5	0.093
(mmol/l)	(1.47 ± 0.35)	(1.40 ± 0.30)	
AST (U/l)	22.8 ± 6.7	23.3 ± 6.5	0.819
ALT (U/l)	22.8 ± 9.7	23.6 ± 9.3	0.759
CPK (U/l)	94 ± 31.3	116 ± 73.8	0.007

*Student’s t-test, p < 0.05.

SD: standard deviation; NTG: post-nitrate; FMD: flow-mediated dilation; EID: endothelium-independent dilation; AST: aspartate transaminase; ALT: alanine transaminase; CPK: creatinine phosphokinase; TG: triglycerides; TC: total cholesterol; LDL-C: low-density lipoprotein cholesterol; HDL-C: high-density lipoprotein cholesterol.

No statistically significant difference was found between the atorvastatin and rosuvastatin groups in respect of baseline transthoracic echocardiographic and brachial artery endothelial function measurements (Table 3). Comparison between the two groups in terms of their effects on non–invasive ultrasonographic brachial artery measurements after one year revealed no statistically significant difference. However, a significant difference was observed in hyperaemia diameter in favour of rosuvastatin (Table 4).

**Table 3:** Statistical comparison between atorvastatin and rosuvastatin groups in terms of baseline brachial artery measurements

*Baseline brachial artery Measurements*	*Atorvastatin Group*	*Rosuvastatin Group*	p-value*
BA basal diameter (mm)	4.01 ± 0.6	4.02 ± 0.5	0.850
BA basal velocity (cm/s )	71.95 ± 14.8	79.32 ± 16.9	0.240
BA hyperaemia diameter (mm)	4.34 ± 0.6	4.43 ± 0.5	0.404
BA hyperaemia velocity (cm/s)	72.21 ± 15.9	73.4 ± 16.3	0.713
BA NTG diameter (mm)	4.6 ± 0.6	4.69 ± 0.5	0.451
BA NTG velocity (cm/s)	68.92 ± 15.4	68.23 ± 15.5	0.833
BA FMD (%)	8.52 ± 3.3	9.71 ± 3.4	0.750
BA EID (%)	15.31 ± 5.1	16.84 ± 5.8	0.159

*Student’s t-test, p < 0.05.

SD: standard deviation; BA: brachial artery; NTG: post-nitrate; FMD: flowmediated dilation; EID: endothelium-independent dilation.

**Table 4:** Rosuvastatin versus atorvastatin in terms of non-invasive test results after 12 months of statin therapy

*Brachial artery measurements after therapy*	*Atorvastatin group (n = 50)*	*Rosuvastatin group (n = 54)*	p-value*
BA basal diameter (mm)	3.87 ± 0.59	4.17 ± 0.54	0.09
BA basal velocity (cm/s)	78.82 ± 13.40	72.55 ± 22.3	0.743
BA hyperaemia diameter (mm)	4.29 ± 0.60	4.60 ± 0.52	0.006
BA hyperaemia velocity (cm/s)	80.03 ± 16.8	71.12 ± 18.5	0.017
BA NTG diameter (mm)	4.78 ± 0.60	4.75 ± 0.52	0.757
BA NTG velocity (cm/s)	71.2 ± 19.45	61.1 ± 16.32	0.323
BA FMD (%)	10.42 ± 3.3	11.75 ± 3.7	0.122
BA EID (%)	16.3 ± 4.83	18.2 ± 5.83	0.115

*Student’s t-test, p < 0.05.

SD: standard deviation; BA: brachial artery; NTG: post-nitrate; FMD: flowmediated dilation, EID: endothelium-independent dilation.

Percentage changes in non–invasive brachial artery measurements after 12 months of treatment were compared between the two groups. A statistically significant difference was found in percentage change in the rosuvastatin group’s brachial artery post–nitrate diameter (p < 0.05). Non–significant changes were found in the basal diameter and hyperaemia velocity in favour of the rosuvastatin group (p = 0.089 and p = 0.088, respectively) (Table 5).

**Table 5:** Changes in brachial artery measurements after 12 months of treatment in the atorvastatin versus rosuvastatin group

*Change in brachial artery measurements after therapy*	*Atorvastatin group (n = 50) Median (25–75%)*	*Rosuvastatin group (n = 54) Median (25–75%)*	p-value*
BA basal diameter (mm)	0.011 (–0.041–0.031)	0.010 (–0.007–0.045)	0.089
BA basal velocity (cm/s)	0.001 (–0.119–0.157)	–0.043 (–0.206–0.378)	0.120
BA hyperaemia diameter (mm)	0.018 (–0.034–0.060)	0.021 (0.011–0.048)	0.644
BA hyperaemia velocity (cm/s)	0.056 (–0.087–0.0347)	–0.012 (–0.168–0.116)	0.088
BA NTG diameter (mm)	0.028 (0.008–0.045)	0.020 (–0.028–0.036)	0.045
BA NTG velocity (cm/s)	–0.004 (–0.167–0.113)	–0.020 (0.0113–0.088)	0.982
BA FMD (%)	0.203 (0.008–0.441)	0.193 (0.049–0.0433)	0.958
BA EID (%)	0.110 (–0.115–0.225)	0.037 (–0.460–0.347)	0.827

*Mann–Whitney U-test, p < 0.05.

BA: brachial artery; NTG: post-nitrate; FMD: flow-mediated dilation; EID: endothelium-independent dilation.

## Discussion

This study revealed that both atorvastatin and rosuvastatin had an effect on baseline lipid values, brachial artery basal diameter and hyperaemia diameter, and FMD and EID measurements. Comparing 12–month non–invasive measurements of atorvastatin and rosuvastatin groups, it was found that the statins had similar effects on endothelial function in the subjects with hyperlipidaemia.

Post–nitrate diameter in the rosuvastatin group was significantly improved at the end of the 12–month treatment compared to baseline values. Endothelial dysfunction is one of the early functional markers of atherosclerosis.^11^,^12^ Preventative measurements should be taken before clinical manifestation of atherosclerotic events. For this reason, detection of early atherosclerotic changes is of great importance in reducing risk factors. Endothelial dysfunction can be detected via FMD, a non–invasive, easily applicable and repeatable method. Studies have demonstrated that FMD was correlated with endothelial function, making it a good marker of endothelial function.^13^,^14^

In the MERCURY I trial, eight–week atorvastatin 20 mg/ day and rosuvastinin 10 mg/day therapies were compared in terms of achieving target LDL–C values of NCEP ATPIII; 80% of the patients in the rosuvastatin group and 74% of those in the atorvastatin group achieved target LDL–C values.^15^ In the SOLAR trial, either atorvastatin 10 mg/day, rosuvastatin 10 mg/ day or simvastatin 20 mg/day was administered as the initial dose for six weeks in 1 634 high–risk patients. The dose was doubled in patients who failed to achieve target value at the end of six weeks. At the end of 12 weeks, target values were achieved with rosuvastatin in 76% of patients, with atorvastatin in 58%, and with simvastatin in 53%.^16^ It is known that atorvastatin 20 mg is pharmacokinetically the same as rosuvastatin 10 mg.^17^ In the present study, LDL cholesterol level decreased with both statins at the end of the 12th month versus baseline, but no statistically significant difference was found between the groups.

The effect of different doses of atorvastatin and rosuvastatin on HDL levels varies according to clinical setting and patient characteristics. The size of the increase is generally more signifcant with lower baseline values. Additionally, the effect is moderate compared to niacin or fibrates. The elevation of HDL level ranges from five to 13%.^18^ In our study, the amount of elevation was not significant, which could have been due to low–dose statin usage or the relatively higher baseline HDL levels of the subjects.

Cardiovascular risk factors such as hyperlipidaemia contribute to endothelial dysfunction, which is the first step in atherogenesis. Although the concurrent presence of hyperlipidaemia and endothelial dysfunction is frequently encountered, the mechanism is unclear. However, oxidised LDL cholesterol is thought to cause endothelial injury. Many studies have demonstrated that endothelium–dependent (flowmediated) dilation is enhanced with increased duration of the endothelium’s exposure to oxidised LDL.^19^–^21^

Kawano et al. demonstrated impaired flow–mediated dilation in an experimental model of acute hyperglycaemia in healthy adults on a fatty diet.^22^ Harrison et al. reported improvement in endothelial function due to decreased cholesterol in the diet.^23^ In studies on statins, the time for endothelial function to improve ranged from hours to months. In earlier studies, improvement in endothelial function with increased NO levels due to statin therapy was observed at the end of a six–month treatment period.^24^,^25^ On the other hand, Marchesi et al. observed remarkable improvement in endothelial function after a two–week atorvastatin therapy in postmenopausal women with hyperlipidaemia.^26^

In the present study, a 22.3 and 30.9% (p = 0.122) increase in FMD was observed in both atorvastatin and rosuvastatin groups, respectively, after 12 months of statin therapy. Improvement in FMD showed no correlation with post–treatment LDL levels. We found however that percentage ΔLDL was well correlated with ΔFMD and ΔEID, which may suggest that statins have a pleiotropic effect independent of their cholesterol–lowering effects. Similarly, ΔLDL was also found to be well correlated with ΔFMD and ΔEID values. In other words, endothelial function was statistically significantly improved at the end of 12–month statin therapy, in parallel with ΔLDL, among patients with hyperlipidaemia.

Although endothelium–independent dilation increased in both groups, the increase was not statistically significantly different. Increased brachial artery basal diameter after 12 months of treatment, which was more pronounced in the rosuvastatin group, reached a value close to the diameter obtained for baseline FMD, due probably to increased NO levels. More prominent increases in FMD and basal diameter in the rosuvastatin group suggest that the NO–secreting effect from the endothelium induced by rosuvastatin was more significant than that by atorvastatin.

There are some limitations of the present study, such as limited patient numbers, as well as not measuring blood NO and asymmetric di–methyl arginine levels due to technical issues. In addition, the technique for measuring FMD depends on the experience of the person carrying it out.

## Conclusion

In this study, both atorvastatin 20 mg/day and rosuvastatin 10 mg/day therapies given to hyperlipidaemic patients for one year provided significant benefits on endothelial function. The data from non–invasive evaluations found that although the favourable effects of rosuvastatin on the endothelium may have been relatively more prominent compared to those of atorvastatin, there was no statistically significant difference.
